# Performance of Large Language Models Under Input Variability in Health Care Applications: Dataset Development and Experimental Evaluation

**DOI:** 10.2196/83640

**Published:** 2026-02-20

**Authors:** Saubhagya Joshi, Monjil Mehta, Sarjak Maniar, Mengqian Wang, Vivek Kumar Singh

**Affiliations:** 1 Library and Information Sciences, School of Communication & Information, Rutgers University, 4 Huntington St, New Brunswick, NJ, 08901, United States, +1 (848) 932-7500; 2 Rutgers Business School, Rutgers University, New Brunswick, NJ, United States; 3 University of North Carolina, Chapel Hill, Chapel Hill, NC, United States

**Keywords:** dataset, error analysis, health informatics, large language models, robustness

## Abstract

**Background:**

Large language models (LLMs) are increasingly integrated into health care, where they contribute to patient care, administrative efficiency, and clinical decision-making. Despite their growing role, the ability of LLMs to handle imperfect inputs remains underexplored. These imperfections, which are common in clinical documentation and patient-generated data, may affect model reliability.

**Objective:**

This study investigates the impact of input perturbations on LLM performance across three dimensions: (1) overall effectiveness in different health-related applications, (2) comparative effects of different types and levels of perturbations, and (3) differential impact of perturbations on health-related terms versus non–health-related terms.

**Methods:**

We systematically evaluate 3 LLMs on 3 health-related tasks using a novel dataset containing 3 types of human-like variations (redaction, homophones, and typographical errors) at different perturbation levels.

**Results:**

Contrary to expectations, LLMs demonstrate notable robustness to common variations, and in more than half of the cases (151/270, 55.92%), the performance was stable or improved. In some cases (38/270, 14.07%), variations resulted in an increased performance, especially when dealing with lower perturbation levels. Redactions, often stemming from privacy concerns or cognitive lapses, are more detrimental than other variations.

**Conclusions:**

Our findings highlight the need for health care applications powered by LLMs to be designed with input variability in mind. Robustness to noisy or imperfect inputs is essential for maintaining reliability in real-world clinical settings, where data quality can vary widely. By identifying specific vulnerabilities and strengths, this study provides actionable insights for improving model resilience and guiding the development of safer, more effective artificial intelligence tools in health care. The accompanying dataset offers a valuable resource for further research into LLM performance under diverse conditions.

## Introduction

### Background

Large language models (LLMs) have revolutionized health care by enhancing patient care, streamlining administrative processes, and advancing medical research. For instance, LLMs are used to analyze patient data for disease management, assist in prior authorization processes by summarizing extensive clinical records, and even support clinical decision-making by providing diagnosis suggestions. Additionally, ambient scribing, where LLMs generate clinical notes from doctor-patient interactions, can significantly reduce the administrative burden on health care providers [1,2].

In such an increasingly LLM-mediated health care ecosystem, understanding LLM robustness—defined as the model’s ability to maintain consistent and accurate performance despite the presence of noise, ambiguity, or variability in input text—is crucial. This includes resilience to typographical errors, domain-specific jargon, abbreviations, and other real-world linguistic variations commonly found in clinical and patient-generated text [3,4]. Such real-world scenarios often invalidate the foundational assumption of many natural language processing systems that rely on clean datasets, especially in health care, where users are frequently tired, sick, or cognitively impaired, and may redact personal information from their queries, either accidentally or intentionally [5]. For instance, related studies suggest that roughly 14% of health search queries contain typos [3].

As a result, evaluations conducted under ideal conditions are not reflective of health care applications, where robustness to input variations is essential. Most existing evaluations focus on nonmedical contexts and do not prioritize robustness, sometimes narrowly construing robustness analysis as attempts to exploit the system for unsafe responses. An often-understudied approach is to assess how everyday users, seeking health information from LLMs, naturally introduce variations and errors in their inputs. Past research has identified several types of errors users make when typing search queries, including phonetic, morphological, orthographic, cognitive, and keyboard layout errors [6]. While there is significant literature on these variations in human input, their impact on LLM performance in health settings remains understudied. In this study, we systematically examine the effects of 3 common types of human variations and levels of perturbation on the performance of LLMs across various health-related tasks. To do so, we also develop and share a novel health–centric dataset with several types of human variations [7,8]. We systematically evaluate the resilience of 3 different LLMs to variations of the original text, particularly in health care contexts. As LLMs continue to be increasingly adopted in health care contexts by professionals and patients alike [9-11], studies like ours are important to find acceptable limits and set guardrails where necessary.

Contrary to expectations, our findings suggest that LLMs are quite robust to common textual perturbations. Furthermore, the impact of these perturbations is far from monotonic—that is, performance does not consistently degrade as input quality worsens. In fact, we observed several instances where model performance improved following certain perturbations. Among the 3 types of variations we studied, redaction—the removal or masking of information, often due to privacy concerns or cognitive lapses [12]—proved to be the most detrimental. This was followed by homophones (words that sound alike but have different meanings or spellings, such as “heal” vs “heel”) and typographical errors (misspellings or accidental keystrokes, such as “diabtes” instead of “diabetes”), which had comparatively milder effects. We hope that our perturbation dataset (Multimedia Appendix 1) and the findings will stimulate more research at the intersection of robustness and health using LLMs. Ensuring that LLMs can handle the imperfect real-world data encountered in health care settings will enhance their reliability and effectiveness, ultimately supporting better patient outcomes.

### Related Work

LLMs have made significant strides in health care applications, such as clinical decision support, medical question answering, and patient education, which constitute 62% of health care use cases [13]. The robustness of LLMs is viewed in terms of benchmarks of efficiency and reliability of models, or user experience metrics of applications [13,14]. Our work emphasizes the impact of user input variations in real-world health care applications.

Prior studies have investigated LLM robustness to word-level and typographical perturbations [15-17] and have also highlighted their capabilities in language error correction [18]. However, evaluating robustness at the intersection of LLMs and health care remains a critical step toward responsible and ethical innovation and adoption in medical settings. This study assesses LLM performance across a range of health-related tasks, including sentiment classification (eg, for patient diarization), medical note classification, and health care–focused question answering [8]. Notably, this study represents one of the earliest systematic efforts to measure and evaluate LLM robustness to human-like input variations within the health care domain.

Typographical errors frequently occur when patients input their symptoms or information, often under stress, sickness, or haste. Homophones are common when mediated by verbal communication (eg, text to speech for doctors’ notes), when written by individuals with limited understanding of medical terms or the English language, or when under cognitive impairments or fatigue [19,20]. Redaction is routinely used to protect sensitive patient information and can also happen under stress or duress [12]. In fact, masking certain words is one of the most common approaches for evaluating LLM model performance. Here, we do not infer intentionality but rather use redaction as a short-hand notation to describe this phenomenon of removing some of the words [18,21]. By focusing on these perturbations, our research aims to provide a targeted and practical assessment of LLM performance in health care settings, making our findings directly interpretable.

### Research Questions

This research aims to provide insights into the following research questions (RQs):

- RQ1: How do perturbations in user input impact the performance of LLMs in health contexts?
- RQ2: What are the relative impacts of different kinds of perturbations on LLMs in health contexts?
- RQ3: What are the relative impacts of perturbations on health-related terms as opposed to other terms on LLM performance in health-related tasks?

## Methods

### Study Design

We designed an experiment with 3 commonly occurring human variations over 3 medically relevant tasks for 3 different LLMs. We introduced various perturbation levels to input texts for each of the 3 tasks and examined the response from each of the LLMs [22,23]. For baseline comparisons, we also measured the LLM responses to the original unperturbed texts.

We evaluated the robustness of LLMs across 3 different medical tasks. The first task is a classification task that required the LLM to identify the sentiment of a given sentence. The second task is also a classification task in which the LLM determined a disease condition from a medical abstract given [7,8,24]. The sentiment task has 2 categories, and medical condition has 3 categories. This design is intended to evaluate the accuracy of the classification using both binary and ternary categories. The third task is a question-and-answer task in which the LLM responded to a question about a medical note provided [7,25].

**Figure 1.**
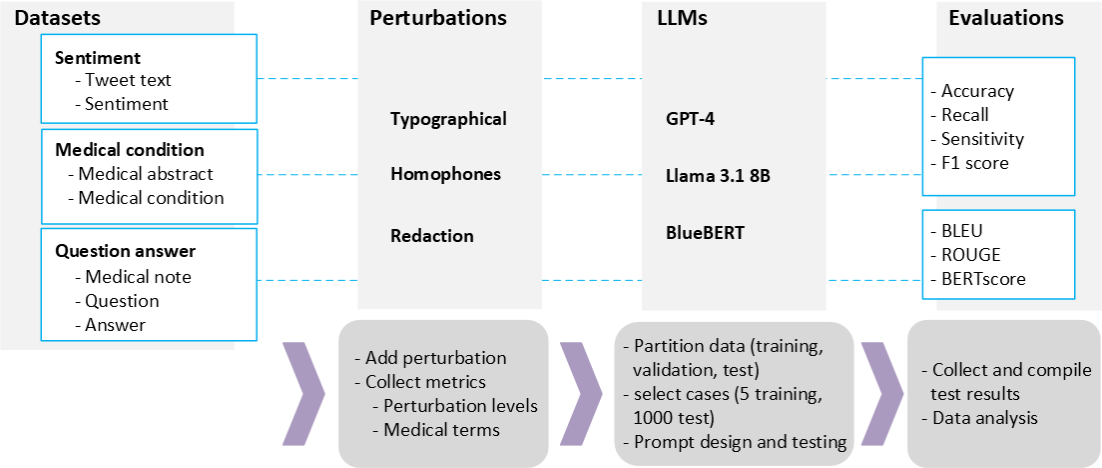
A summary of the experimental design across dimensions (perturbation types, perturbation levels, tasks, and large language models [LLMs]) and the workflow leading from dataset generation, collection of data, pre-processing, and analysis. BERT: Bidirectional Encoder Representation from Transformers; BLEU: Bilingual Evaluation Understudy; ROUGE: Recall-Oriented Understudy for Gisting Evaluation.

The experiment workflow is shown in Figure 1. The input data obtained from each of the datasets is perturbed and packaged within the prompt. This prompt is sent to the LLM via an application programming interface (API), and the responses are recorded. These responses are compared and evaluated based on the ground truth available in the original database. The settings for each of the LLMs are summarized in part C in Multimedia Appendix 2.

### Perturbations

End users and patients often turn to general-purpose tools, such as search engines and publicly available chatbots—even when more specialized health care models exist—for health-related information-seeking and decision support [5,26]. To reflect this real-world usage pattern, we selected both general-purpose and domain-specific LLMs for evaluation, with a focus on their utility in health-related tasks.

We perturbed the input text provided to the LLMs using 3 techniques intended to simulate typical human-based perturbations: first, we introduced typographical errors; second, we used homophones to replace words in the text (eg, disease vs decease); and third, we removed some words from the text [21]. The examples of such perturbations are shown in Table 1.

**Table 1.** Perturbation types used in the study with specific examples for each perturbation that is applied to the original sample.

Original	How did^a^ voxelotor affect^b^ the patient’s scleral icterus^c^ and overall quality of life^c^ in^a^ the given discharge^b^ summary?
Typographical	How did voxelotor affec^b^ the patient’s scleral icterus and overall quality of life in the given dicsharge^b^ summary?
Homophone	How deed^a^ voxelotor affect the patient’s scleral icterus and overall quality of life inn^a^ the given discharge summary?
Redaction	How did voxelotor affect the patient’s scleral and overall quality of in the given discharge summary?

a Homophone.

b Typographical error.

c Redaction.

### Perturbation Levels

Different levels of perturbations elicit different responses from LLMs. Each perturbation technique is applied as a percentage of the textual data to systematically investigate the models’ robustness. In this study, we use 3 levels of perturbation: low, medium, and high. The operationalization for each type of perturbation is described below.

### Typographical Errors

Perturbations are introduced as a percentage of the total number of characters. We test typographical errors at 10%, 30%, and 50%, which we refer to as low, medium, and high perturbation levels, respectively.

### Homophone Substitutions

These perturbations are applied as a percentage of the total number of words, specifically at 10%, 20%, and 30%, which we refer to as low, medium, and high, respectively. Given that not all words have homophones, these lower percentages are realistic for evaluating the effects of homophone confusion in medical queries. We consider the first level of perturbation (eg, 10%) in each category to be low and the next 2 levels to be medium and high, respectively. By systematically varying these perturbation levels, we aim to understand how LLMs handle common errors in medical contexts, thereby assessing their practical utility and reliability in real-world health care applications.

### Redactions

Perturbations are implemented as a percentage of the total number of words. We examine the impact of word redaction at the 10%, 30%, and 50% levels, simulating scenarios where critical information might be partially obscured or omitted in medical documentation. Similar to typographical errors, we refer to these levels as low, medium, and high.

### Dataset Creation

We selected 3 datasets as the tasks for this study: tweet emotions [27], medical abstracts [28], and question answering [29]. Our perturbation process targets semantically valid words, which we define based on their part-of-speech tags. Specifically, we consider adjectives, adverbs, verbs, and nouns as valid words for perturbation. Since sentences vary in the number of valid words they contain, we adjusted the perturbation to ensure that a specific percentage of words in each sentence is perturbed while only targeting valid words. For example, in a sentence containing 10 words, of which 5 are valid, a 10% perturbation rate corresponds to perturbing 1 valid word, effectively 20% of the valid words in that sentence.

### Perturbation Techniques

After identifying the words to perturb, we applied one of the following transformations:

- Typographical perturbations: insertion, deletion, substitution, or transposition of letters in the chosen word. We randomly perturbed 20%-50% of the letters in a word.
- Homophone substitution: used the Datamuse API to retrieve potential homophones [22]. For each valid word, the first available homophone replaced the original word
- Redaction: removal of specified percentage of the selected valid words

After applying perturbations, we retained only examples that could generate all specified perturbation percentages (eg, 10%, 30%, 50%). These perturbed representations are combined into a new dataset called Health-LLM-Perturbation Dataset that we will be sharing with the community to support similar research by others.

### Training and Testing Splits

For generative language model experiments, we split the data as follows:

- Prompt tuning: 20% of the dataset
- Testing: randomly selected 1000 samples from the 80%
- Validation: remaining of 80% after test set extraction

For Bidirectional Encoder Representation from Transformers (BERT)–based language model experiments, we randomized the dataset, excluding the same 1000 test samples, and divided the remaining data into an 80% training set and a 20% validation set. In question-answer experiments, even if a medical note had multiple associated questions in the original dataset, we selected only one of those questions to include in our dataset. Training, validation, and test sets were split based on unique medical notes to ensure no overlap between the sets.

### Design of Prompt

The conversational prompt may take various forms depending on the medical task. We adopted a few-shot (n=5) prompting approach in this work. Few-shot prompting is a technique to guide LLMs to perform specific tasks by providing a few examples (or “shots”) within the prompt. The exact prompts for each of the tasks are given in part A of Multimedia Appendix 2. The basic structure of a 5-shot prompt consisted of a role assignment, 5 examples, specific instructions, input data, and other instructions (if necessary). In the case of classification tasks, this basic structure was followed. For the question-and-answer tasks, we additionally instructed the LLM to provide an answer based only on the medical note provided. With an API call to the LLM, each data text was tested within its own session. However, when testing manually, the LLMs sometimes responded based on previous prompts, especially if the medical notes were similar. As a precautionary measure, to ensure separation across medical cases, specific instructions were included to prevent LLM from being contaminated by short-term memory. To improve the accuracy of the response, we instructed the LLM to provide a short justification, and in case no justification could be provided, the LLM was instructed to respond accordingly. Asking the LLM to provide an explanation for the response helped the LLM provide a more accurate answer [23].

### Metrics for Evaluation

For classification tasks, such as sentiment and medical condition, 4 metrics were used for evaluation: accuracy, recall, precision, and F1. For the question-answer task, distance measures such as BLEU (Bilingual Evaluation Understudy) [30], ROUGE (Recall-Oriented Understudy for Gisting Evaluation) [31], and BERTScore (Bidirectional Encoder Representation from Transformers) [32] were used [33]. Although the 3 measures were used, only one of the measures would be used for analysis because averaging the 3 different measures would not be meaningful and introduce noise. The purpose of collecting data was to see if the patterns were different for the 3 measures. For researchers interested in any of the 3 measures, we make the results available in Tables S7 and S8 in Multimedia Appendix 2.

We evaluated the performance in terms of variation from the original average. For ease of interpretation, we split them into the following 4 categories [34-36]:

- Increase: performance increases (rather than decreases) with perturbations
- Stable: performance decreases less than 5% of original
- Decrease: performance decreases more than 5% but less than 50%
- Catastrophic drop: performance decreases less than 50% of original

We used the statistical package of Microsoft Excel to perform statistical tests. To examine the effect of perturbations RQ1, we aggregated the results and determined the proportions of the levels of impact of perturbations. Then, we performed a Pearson chi-squared test to check if perturbation effects were independent across tasks and LLMs. To examine the relative impact of perturbations across dimensions RQ2, we conducted repeated measures ANOVA across tasks, perturbations, perturbation levels, and LLMs, which is reported in Table S9 in Multimedia Appendix 2. In order to strengthen our numerical analysis by assuming nonnormal data, we conducted a nonparametric Friedman chi-square test [37] across groups for each dimension, followed by post hoc tests using pairwise Conover tests with the Bonferroni correction. To examine the effect of perturbations on medical terms RQ3, we compiled the proportions of perturbed text that were medical terms and then evaluated the effects.

### Ethical Considerations

All study procedures complied with OpenAI’s usage policies and ethical guidelines. No personal or sensitive information was used or generated during the research, and all data were securely stored with access restricted to authorized research personnel. No human subject was involved, and all data used were based on publicly available sources. Therefore, an ethics approval was not sought. The overarching goal of this study is to contribute to the development of health LLMs that are safe and equitable across input variability. By systematically evaluating the impact of different levels of input variability on health LLMs, this exploratory work aims to support more inclusive and socially beneficial AI systems.

## Results

### RQ1: Robustness to Perturbations

The performance observed for various perturbations for each task and at each level of perturbation is shown in Tables S1-S8 in Multimedia Appendix 2. In Table 2, we summarize the findings by groups as defined in the *Methods* section. For the question-answer task, all 3 measures of BLEU, ROUGE, and BERTScore had similar patterns. For purposes of analysis, we used ROUGE because these values were approximately between the other 2 measures and would greatly simplify our analysis without unnecessary complexity.

**Table 2.** The effect of perturbations across different tasks for each large language model (LLM) is shown as 4 levels of robustness ranging from minimal (increase, stable) to decrease and catastrophic^a^.

LLMs per task	Increase	Stable	Decrease	Catastrophic	Total
Total, n (%)	38 (14.07)	113 (41.85)	104 (38.52)	15 (5.56)	270 (100)
Sentiment, n					
GPT^b^	6	2	19	9	36
BlueBERT^c^	0	16	20	0	36
Llama	0	19	14	3	36
Medical abstract, n					
GPT	13	15	5	3	36
BlueBERT	0	28	8	0	36
Llama	0	15	21	0	36
Question answer, n					
GPT	15	1	11	0	27
Llama	4	17	6	0	27

a These robustness bins allow the results to distinguish catastrophic effects from other robustness bins. This interpretation provides a practical approach to evaluating robustness according to existing literature. LLMs are robust to perturbations because only about 6% (4/72) of the perturbed cases resulted in catastrophic failure.

b GPT: generative pretrained transformer.

c BERT: Bidirectional Encoder Representation from Transformers.

Our first observation was that the impact of perturbations was far from uniform or monotonic. While the performance on average decreased with perturbation, some notable findings are as follows:

- In 38 (14.07%; nearly 1 in 7) out of 270 scenarios, the performance of LLMs improved rather than decreased with perturbations.
- In more than 40% (113/270, 41.85%) of the scenarios, the performance of LLMs remained stable. Thus, in more than half (151/270, 55.92%) of the cases, the performance was stable or improved.
- In 15 (5.56%) out of the 270 scenarios, the performance of LLMs dropped to catastrophically poor levels. Most (12/15) of these catastrophic drops occurred when using generative pretrained transformer rather than BlueBERT or Llama.

The chi-squared tests across LLMs (*χ*^2^_6_=81.25, *P*<.001) and tasks (*χ*^2^_6_=44.74, *P*<.001) were both significant.

Next, we compared the impact of perturbations across the various dimensions by examining the variance of means. We performed a repeated measures ANOVA across the various dimensions, all of which were significant (part D of Multimedia Appendix 2, Table 1), and then followed up with post hoc *t* tests. In order to strengthen the numerical analysis in nonnormal conditions, we performed separate Friedman [37] chi-squared (*Q*) tests across the groups for perturbation types (Friedman *χ*^2^_2_=8.66, *P*=.01), for LLMs (Friedman *χ*^2^_2_=18.428, *P*<.001), for task types (Friedman *χ*^2^_2_=7, *P*=.03), and for perturbation levels (Friedman *χ*^2^_2_=26.547, *P*<.001). Since all 4 tests are significant, we performed post hoc tests using pairwise tests using the Conover with Bonferroni correction. The results of the Friedman test and post hoc tests are given in Table 3. Two of the effects show no effect, and the rest are either medium or large effects.

**Table 3.** Robustness to perturbations across repeated measures of each dimension (perturbation types, perturbation levels, tasks, and large language models [LLMs]) is examined using nonparametric Friedman chi-square tests over groups showing significant variation across groups in each dimension^a^.

Group 1	Group 2	Pairwise Conover tests using Bonferroni correction			Friedman *χ*^2^ test (*df*)	*P* value
		*t* value	*P* value	Effect size (*r*)		
Perturbation types					8.66 (2)	.01
Typographical	Homophone	0.006	>.99	0.0009		
Homophone	Redaction	0.88	.02	0.128		
Redaction	Typographical	0.877	.03	0.127		
Perturbation levels					26.547 (2)	<.001
Original	Low	0.84	.002	0.099		
Low	Medium	1.45	<.001	0.17		
Medium	Original	2.29	<.001	0.27		
Tasks					7 (2)	.03
Sentiment	Medical abstract	1.3	.46	0.2		
Medical abstract	Question answer	3.1	.02	0.51		
Question answer	Sentiment	2.003	.46	0.309		
LLMs					18.428 (2)	<.001
GPT^b^	BlueBERT^c^	4.32	<.001	0.67		
BlueBERT	Llama	1.22	>.99	0.19		
Llama	GPT	3.11	<.001	0.48		

a Pairwise post hoc tests across groups using Conover tests with the Bonferroni correction showing magnitude, significance, and effect size for the ranked tests (*r*). The results show significant differences between pairs in each dimension with medium-to-large effect size. Specifically, (1) robustness to typographical errors and homophones is similar, (2) perturbation levels have significant effects, (3) robustness between medical abstract and question and answer is significantly different with large effect, and (4) robustness across large language models (generative pretrained transformer vs Llama and generative pretrained transformer vs BlueBERT) is significantly different with a large effect.

b GPT: generative pretrained transformer.

c BERT: Bidirectional Encoder Representation from Transformers.

### RQ2: Relative Impact of Perturbations Across Dimensions

**Figure 2.**
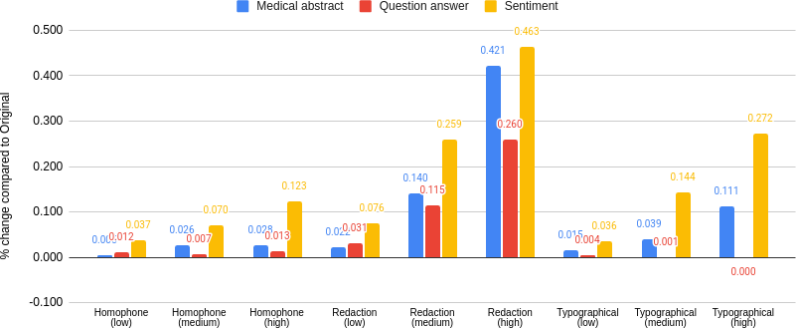
A color-coded bar chart showing the effect of 3 levels of perturbation (low, medium, and high levels) as a percentage of change for each task (blue color for medical abstract, red color for question answer, and yellow for sentiment) compared to the original unperturbed data across different types of perturbation (homophone, redaction, and typographical).

Here, we study how the impact of perturbation varies across types of perturbation. As shown in Figure 2, all the tasks are most prominently affected by redaction. Although the question-answer task is affected more by homophone perturbations than by typographical errors, both sentiment and medical abstract tasks are affected more by typographical errors than by homophones.

Table 4 shows that typographical errors and homophone-based perturbations have relatively limited impact, whereas redaction-based perturbations lead to a much more pronounced degradation in performance, especially in terms of the increased number of catastrophic responses.

**Table 4.** The effect of perturbation types is shown as 4 levels of robustness ranging from minimal (increase, stable) to decrease and catastrophic^a^.

Perturbation types	Increase	Stable	Decrease	Catastrophic	Total
Total, n (%)	5 (6.9)	35 (48.6)	28 (38.9)	4 (5.6)	72 (100)
Typographical, n	4	11	9	0	24
Homophone, n	1	16	7	0	24
Redaction, n	0	8	12	4	24

a These robustness bins allow the results to distinguish catastrophic effects from other robustness bins. This interpretation provides a practical approach to evaluating robustness according to existing literature. The results show that large language models are, in general, robust to low-to-medium level of perturbations for typographical errors and homophones. However, medium levels of redactions can trigger large language models to catastrophic results.

### RQ3: Differences in the Impact of Medical Versus Nonmedical Terms

Next, we consider the relative impact of the perturbations on a medical term versus a nonmedical term in the user prompt. The trends varied across settings and were not obvious in aggregate terms. To explore the nuanced effect of perturbation on medical terms, we examined the performances across different dimensions.

**Figure 3.**
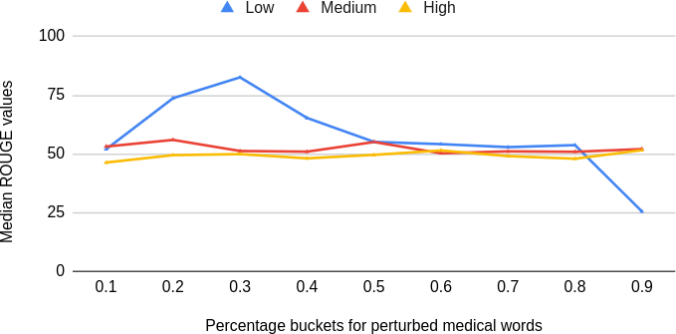
Variation in median ROUGE (Recall-Oriented Understudy for Gisting Evaluation) values observed across buckets of percentage of medical words perturbed for different perturbation levels (blue color for low perturbation level, red for medium perturbation level, and yellow for high perturbation level) showing higher variability for low perturbation levels and a decreasing trend with higher ratio of medical words being perturbed.

Figure 3 shows some variation in median ROUGE scores when measured by the percentage of perturbed medical terms in the LLM prompt. For low perturbations (10% perturbation level), there is visible variation. However, for medium and high levels of perturbations, the variation is not apparent. In general, the performance shows a decreasing trend with a higher ratio of medical words being perturbed.

We also conducted preliminary error analysis to understand the patterns in the data and interpret some of the macro observations identified above. This analysis was undertaken with the manual inspection of samples used in the classification of medical conditions.

At a macro level, consistent with the findings in Table 2, we found that minor perturbations often did not impact the model’s performance. In fact, in some cases, performance improved slightly at lower perturbation levels. This robustness was particularly evident when perturbations occurred in words that were less critical to the classification task, such as prepositions (“in” or “off”) that did not alter the medical context or disease description. These changes typically had negligible effects on the output, and the performance remained stable.

However, when perturbations targeted key medical terms critical for classification, the model’s performance declined, as expected. Interestingly, instances arose where both the LLM and a human nonexpert could become confused between 2 similar medical conditions, especially when there was overlapping information in the description. In such cases, if the perturbation affected the distractor (ie, the secondary, less relevant condition), the performance improved as the model’s focus shifted back to the correct classification. Conversely, when the perturbation impacted the primary condition, the degradation in performance was not as severe at low perturbation levels, as the LLM was already likely to be uncertain due to the inherent ambiguity of the input.

The following medical abstract is classified as a “Digestive System Disease” as per the ground truth in the dataset:

- Classification: 2
- Medical abstract: Carcinoma of the gallbladder. Gallbladder cancer remains difficult to diagnose preoperatively. However, recent work suggests that ultrasound may be effective. Gallbladder cancer remains highly lethal despite aggressive therapy. Extension of the disease beyond the mucosa predicts a poor chance of long-term survival.

Even without any perturbation, the LLM incorrectly classified this as “Neoplasm” and failed to provide adequate justification for the misclassification. Notably, when key medical terms such as “carcinoma” or “cancer” were perturbed, the LLM correctly classified the abstract as a “Digestive System Disease.” This observation supports the hypothesis that low-level perturbations can impact distractors and potentially enhance LLM performance in cases where multiple potential medical conditions exist.

## Discussion

### Principal Findings

Our study systematically evaluated the robustness of LLMs to different types of perturbations in health care contexts. The results reveal several insights into how LLMs perform under various conditions, which are discussed in relation to our RQs.

#### RQ1: Robustness to Perturbations

Perturbations affect LLM performance in nonuniform, nonmonotonic ways. While performance generally declines with increased perturbations, 1 in 7 cases showed improvement, and over half remained stable or improved, indicating resilience to certain input variations. However, occasional severe drops, especially with ChatGPT, underscore the need for stronger robustness measures. These findings highlight the importance of testing LLMs under realistic conditions for reliable health care use.

#### RQ2: Relative Impact of Different Perturbations

The effect of perturbations varies by task and type. Redaction had the most detrimental impact, as it disrupts context more than typos or homophones. This is a significant concern in health care, where patients may redact data for privacy or due to cognitive lapses. Such disruptions can reduce accuracy in clinical and educational applications, emphasizing the need for LLMs resilient to missing information. Homophones most affected question-answer tasks, while typographical errors impacted sentiment and medical condition tasks more. This indicates the need for task-specific training to enhance robustness.

#### RQ3: Medical vs Nonmedical Terms

At low perturbation levels, LLMs were more sensitive to medical term disruptions, underscoring the importance of precise medical language processing. At higher perturbation levels, performance degraded uniformly across term types, suggesting that excessive noise compromises overall model reliability. Ensuring robustness to both medical and general vocabulary is therefore essential for health care applications.

### Implications for Health Informatics

The implications of our findings for health informatics are substantial. Ensuring the robustness of LLMs to real-world variations in input can significantly enhance their effectiveness and reliability in health care applications. This robustness is crucial for clinical decision support, patient education, and medical question answering, where accurate and dependable responses are paramount. While the general trend of robustness is encouraging, the potential for catastrophic drops is alarming. The variegated impact of different types of perturbations on performance in different settings can also inform the design of future health LLM systems on key aspects to prioritize building robust health LLMs. An incidental implication of our findings from error analysis of medical terms is the importance of medical experts in medical diagnosis and the use of LLMs as auxiliary support tools in health care. Further, the results suggest that designing ensemble methods that can combine the responses of multiple similar queries (some of which are perturbed versions of the original query) can be a useful pathway to increase LLM response accuracy. Further, our contribution to sharing a novel health-centric dataset with different types of human errors and levels of perturbation provides a valuable resource for further research. By making this dataset available, we aim to stimulate more research at the intersection of robustness and health LLMs, ultimately contributing to better patient outcomes and more efficient health care systems.

In alignment with the use cases of LLMs supporting clinical decision-making [10], our study has given empirical evidence of robustness levels in response to perturbation types and perturbation levels for LLMs across a variety of medical tasks. Sentiment analysis is an important component of clinical decision-making for both clinicians [38] and patients [39,40]. Autonomous systems that involve triaging patients and screening referrals similarly make use of question-answer tasks and medical condition summaries. This study also adds to the literature on the axis of human–artificial intelligence synergy and bias and data issues, which are 2 of the key dimensions of health care AI literature [41]. Beyond accuracy, error-tolerant prompts, and redaction-aware design, safe and reliable health care LLMs are contingent upon contextual cues, safety guardrails, and regulatory checks in routine care.

### Limitations

While our study provides valuable insights into LLM robustness in health care contexts, it has several limitations that we plan to address in future work. First, the perturbations used—typographical errors, homophones, and redactions—were synthetically generated to simulate common real-world variations. Although representative and a useful starting point, these do not capture the full range of linguistic and contextual variability seen in actual clinical and patient-generated text. In future work, we will expand the scope of perturbations to include additional forms, such as abbreviations, syntactic reordering, and multilingual input. We also aim to incorporate real-world user data to better reflect the diversity of health care communication. Additionally, our current evaluation focused on 3 LLMs; extending this analysis to a broader set of models will help generalize our findings across architectures and domains.

We are also actively exploring practical mitigation strategies for real-world deployment, including interactive input filters and automated detection of high-risk queries. These efforts aim to enhance the safety and reliability of LLM-powered health care tools. Importantly, we are releasing our novel perturbation dataset to the research community, providing a valuable resource for benchmarking and advancing robustness in health-related natural language processing applications.

Despite the limitations, this study represents a significant step forward in understanding how LLMs perform under realistic input conditions. By identifying key vulnerabilities and sharing tools to address them, we aim to catalyze the development of more resilient, equitable, and trustworthy AI systems in health care.

### Conclusion

This study provides a comprehensive evaluation of the robustness of LLMs to multiple perturbations in health care contexts, specifically typographical errors, homophones, and redactions, revealing differing levels of resilience across health-related tasks. While LLMs exhibit adaptability to some input variations, redaction-based perturbations significantly impair their contextual understanding. These findings emphasize the necessity of robust evaluation frameworks that mirror real-world input variations to ensure the reliability of LLMs in applications, such as clinical decision support, patient education, and medical question answering. Our contribution to a health care–specific dataset with diverse perturbations aims to advance research by fostering the development of more resilient LLMs. Future research should explore additional perturbation types, include a broader range of LLMs, and incorporate diverse user interactions to better simulate real-world scenarios, ultimately driving the creation of dependable and impactful health informatics systems.

## Supplementary material

10.2196/83640 Multimedia Appendix 1 Input perturbation dataset.

10.2196/83640 Multimedia Appendix 2 Prompts, results, settings, and ANOVA results with post hoc tests.

## Abbreviations

API: application programming interface
BERT: Bidirectional Encoder Representation from Transformers
BLEU: Bilingual Evaluation Understudy
LLM: large language model
ROUGE: Recall-Oriented Understudy for Gisting Evaluation
RQ: research question

## References

[1] Hargittai E (2006). Hurdles to information seeking: spelling and typographical mistakes during users’ online behavior. J Assoc Inf Syst.

[2] Hasan S, Heger C, Mansour S Spelling correction of user search queries through statistical machine translation.

[3] Crowell J, Zeng Q, Ngo L, Lacroix EM (2004). A frequency-based technique to improve the spelling suggestion rank in medical queries. J Am Med Inform Assoc.

[4] Klotzman V (2023). Engineering Mathematics and Artificial Intelligence Foundations, Methods, and Applications.

[5] Shahsavar Y, Choudhury A (2023). User intentions to use ChatGPT for self-diagnosis and health-related purposes: cross-sectional survey study. JMIR Hum Factors.

[6] Cox D, Cox JG, Cox AD (2017). To err is human? How typographical and orthographical errors affect perceptions of online reviewers. Comput Human Behav.

[7] Blagec K, Dorffner G, Moradi M, Ott S, Samwald M A global analysis of metrics used for measuring performance in natural language processing.

[8] Kané H, Kocyigit Y, Ajanoh P, Abdalla A, Coulibali M (2019). Towards neural language evaluators. arXiv.

[9] Lee P, Bubeck S, Petro J (2023). Benefits, limits, and risks of GPT‑4 as an AI chatbot for medicine. N Engl J Med.

[10] Magrabi F, Ammenwerth E, McNair JB (2019). Artificial intelligence in clinical decision support: challenges for evaluating AI and practical implications. Yearb Med Inform.

[11] Tangsrivimol JA, Darzidehkalani E, Virk HUH (2025). Benefits, limits, and risks of ChatGPT in medicine. Front Artif Intell.

[12] Bullement A, Taylor M, McMordie ST, Waters E, Hatswell AJ (2019). NICE, in confidence: an assessment of redaction to obscure confidential information in single technology appraisals by the National Institute for Health and Care Excellence. Pharmacoeconomics.

[13] Tam TYC, Sivarajkumar S, Kapoor S (2024). A framework for human evaluation of large language models in healthcare derived from literature review. NPJ Digit Med.

[14] Nucci A (2026). LLM evaluation: key metrics and frameworks. Aisera.

[15] Dhole KD, Gangal V, Gehrmann S (2022). NL-augmenter: a framework for task-sensitive natural language augmentation. arXiv.

[16] Liang P, Bommasani R, Lee T (2022). Holistic evaluation of language models. arXiv.

[17] Wang H, Ma G, Yu C (2023). Are large language models really robust to word-level perturbations?. arXiv.

[18] Singh A, Singh N, Vatsal S (2024). Robustness of large language models to perturbations in text. arXiv.

[19] Lee EB, Heo GE, Choi CM, Song M (2022). MLM-based typographical error correction of unstructured medical texts for named entity recognition. BMC Bioinformatics.

[20] Yu T, Liu X, Ding L, Chen K, Tao D, Zhang M Speech sense disambiguation: tackling homophone ambiguity in end-to-end speech translation.

[21] Wang Y, Zhao Y (2024). RUPBench: benchmarking reasoning under perturbations for robustness evaluation in large language models. arXiv.

[22] (2025). Datamuse API. Datamuse.

[23] Gade K, Geyik S, Kenthapadi K, Mithal V, Taly A Explainable AI in industry: practical challenges and lessons learned.

[24] Fabbri AR, Kryściński W, McCann B, Xiong C, Socher R, Radev D (2021). SummEval: re-evaluating summarization evaluation. Trans Assoc Comput Linguist.

[25] He Z, Bhasuran B, Jin Q (2024). Quality of answers of generative large language models versus peer users for interpreting laboratory test results for lay patients: evaluation study. J Med Internet Res.

[26] Wang L, Wan Z, Ni C (2024). Applications and concerns of ChatGPT and other conversational large language models in health care: systematic review. J Med Internet Res.

[27] Gupta P (2025). Emotion detection from text. Kaggle.

[28] (2025). Sebischair/medical‑abstracts‑TC‑corpus. GitHub.

[29] (2023). Starmpcc/asclepius. GitHub.

[30] Papineni K, Roukos S, Ward T, Zhu WJ BLEU: a method for automatic evaluation of machine translation.

[31] Lin CY ROUGE: a package for automatic evaluation of summaries. https://aclanthology.org/W04-1013/.

[32] (2020). BERTScore: evaluating text generation with BERT. OpenReview.

[33] Schopf T, Braun D, Matthes F Evaluating unsupervised text classification: zero-shot and similarity-based approaches.

[34] Braiek HB, Khomh F, Lorenzi M, Zuluaga MA (2025). Trustworthy AI in Medical Imaging.

[35] Dünkel O, Jesslen A, Xie J (2025). CNS-bench: benchmarking image classifier robustness under continuous nuisance shifts. arXiv.

[36] Kim H, Lee W, Lee J (2020). Understanding catastrophic overfitting in single-step adversarial training. arXiv.

[37] Sheldon MR, Fillyaw MJ, Thompson WD (1996). The use and interpretation of the Friedman test in the analysis of ordinal-scale data in repeated measures designs. Physiother Res Int.

[38] Hah H, Goldin DS (2021). How clinicians perceive artificial intelligence‑assisted technologies in diagnostic decision making: mixed methods approach. J Med Internet Res.

[39] Shankar R, Yip A (2025). Transforming patient feedback into actionable insights through natural language processing: knowledge discovery and action research study. JMIR Form Res.

[40] Shankar R, Xu Q, Bundele A (2025). Patient voices in dialysis care: sentiment analysis and topic modeling study of social media discourse. J Med Internet Res.

[41] Ogut E (2025). Artificial intelligence in clinical medicine: challenges across diagnostic imaging, clinical decision support, surgery, pathology, and drug discovery. Clin Pract.

