# Supplementary material for: Performance of Large Language Models Under Input Variability in Health Care Applications: Dataset Development and Experimental Evaluation
Source: JMIR AI. 2026 Feb 20;5:e83640. doi: 10.2196/83640 (PMC12923095; doi:10.2196/83640)
Supplement: Multimedia Appendix 2 [file ai-v5-e83640-s002.pdf]

SUPPLEMENTARY DOCUMENT: APPENDIX

Performance of Large Language Models Under Input Variability in Healthcare Applications: Dataset Development and Experimental Evaluation

Saubhagya Joshi, Monjil Mehta, Sarjak Maniar, Menqian Wang, and Vivek K. Singh

A. Prompts

A.1 Task: sentiment

```
[{'role': 'system', 'content': 'You are a sentiment
classification expert who classifies twitter sentiments
into one of [<sentiment categories>]'},
{"role": "user", "content": <tweet example 1>},
{"role": "assistant", "content": <sentiment for
example 1>}
{"role": "user", "content": <tweet example 2>},
{"role": "assistant", "content": <sentiment for
example 2>}
{"role": "user", "content": <tweet example 3>},
{"role": "assistant", "content": <sentiment for
example 3>}
{"role": "user", "content": <tweet example 4>},
{"role": "assistant", "content": <sentiment for
example 4>}
{"role": "user", "content": <tweet example 5>},
{"role": "assistant", "content": <sentiment for
example 5>},
{'role': 'user', 'content': 'Please categorize
the following tweet only into one of [<sentiment
categories>] and justify with one or two
    sentences. If you cannot justify, respond
 with "Unknown" and no other text:\n <test prompt>}]
```

A.2 Task: med abstract

```
[{'role': 'system', 'content': 'You are a medical expert
who categorizes medical notes into one of [<condition names>]'},
{"role": "user", "content": <medical abstract 1>},
{"role": "assistant", "content": <condition for medical
abstract 1>}
{"role": "user", "content": <medical abstract 2>},
{"role": "assistant", "content": <condition for medical
abstract 2>}
{"role": "user", "content": <medical abstract 3>},
{"role": "assistant", "content": <condition for medical
abstract 3>}
{"role": "user", "content": <medical abstract 4>},
{"role": "assistant", "content": <condition for medical
abstract 4>}
{"role": "user", "content": <medical abstract 5>},
{"role": "assistant", "content": <condition for medical
abstract 5>},
{'role': 'user', 'content': 'Please categorize the
following medical note only into one of [<condition
names>] and
    justify your answer in one or two sentences. If
 you cannot justify, please answer with "Unknown" and
    no other text:\n' <test case>}]
```

A.3 Task: QnA

```
[{'role': 'system', 'content': 'You are a medical expert
who answers questions based on the given "Medical Note":'},
{"role": "user", "content": "Medical Note for patient
<patient_id 1> begins:\n
    <medical note 1>
    \nMedical Note for <patient_id 1> ends.\n
    Please answer the following question based on the
 given "Medical Note" for patient <patient_id 1>:\n
    <Question for medical note 1>},
{"role": "assistant", "content": <answer for medical
note 1>}
{"role": "user", "content": "Medical Note for patient
<patient_id 2> begins:\n
    <medical note 2>
    \nMedical Note for <patient_id 2> ends.\n
    Please answer the following question based on the
 given "Medical Note" for patient <patient_id 2>:\n
    <Question for medical note 2>},
{"role": "assistant", "content": <answer for medical
note 2>}
{"role": "user", "content": "Medical Note for patient
<patient_id 3> begins:\n
    <medical note 3>
    \nMedical Note for <patient_id 3> ends.\n
    Please answer the following question based on the
 given "Medical Note" for patient <patient_id 3>:\n
    <Question for medical note 3>},
{"role": "assistant", "content": <answer for medical
note 3>}
{"role": "user", "content": "Medical Note for patient
<patient_id 4> begins:\n
    <medical note 4>
    \nMedical Note for <patient_id 4> ends.\n
    Please answer the following question based on the
 given "Medical Note" for patient <patient_id 4>:\n
    <Question for medical note 4>},
{"role": "assistant", "content": <answer for medical
note 4>}
{"role": "u ser", "content": "Medical Note for patient
<patient_id 5> begins:\n
    <medical note 5>
    \nMedical Note for <patient_id 5> ends.\n
    Please answer the following question based on the given
"Medical
 Note" for patient <patient_id 5>:\n
    <Question for medical note 5>},
{"role": "assistant", "content": <answer for medical note 5>}
```

```
{'role': 'user', 'content': 'Medical Note for patient <patient_id
for test case> begins:\n
<medical note for test case>
\nMedical Note for <patient_id for test case>  ends.\n
Please provide a short answer the following  question based on
the
given "Medical Note" for patient <patient_id for test case>:\n
Question for medical note for test case>}]
```

B. Results Summary

B.1. Task: Sentiment

B.1.1 GPT4o

Table S1: Average metrics of GPT4o responses to perturbations

		Typographical			Homophone			Removal		
	**orig**	**0.1**	**0.3**	**0.5**	**0.1**	**0.2**	**0.3**	**0.1**	**0.3**	**0.5**
**accuracy**	0.639	0.571	0.375	0.212	0.581	0.495	0.424	0.484	0.136	0.025
**precision**	0.918	0.933	0.912	0.873	0.929	0.935	0.94	0.923	0.959	0.85
**recall**	0.639	0.571	0.375	0.212	0.581	0.495	0.424	0.484	0.136	0.025
**f1**	0.746	0.697	0.51	0.318	0.705	0.633	0.567	0.618	0.222	0.048

B.1.2 LLama 3.1 8B

Table S2: Average metrics of LLama 3.1 8B responses to perturbations

		Typographical			Homophone			Removal		
	**orig**	**0.1**	**0.3**	**0.5**	**0.1**	**0.2**	**0.3**	**0.1**	**0.3**	**0.5**
**accuracy**	0.822	0.804	0.776	0.688	0.798	0.789	0.752	0.788	0.67	0.286
**precision**	0.841	0.831	0.814	0.775	0.826	0.82	0.803	0.821	0.774	0.678
**recall**	0.822	0.804	0.776	0.688	0.798	0.789	0.752	0.788	0.67	0.286
**f1**	0.828	0.814	0.794	0.727	0.807	0.802	0.771	0.801	0.713	0.399

B.1.3 BlueBERT

Table S3: Average metrics of BlueBERT responses to perturbations

		Typographical			Homophone			Removal		
	**orig**	**0.1**	**0.3**	**0.5**	**0.1**	**0.2**	**0.3**	**0.1**	**0.3**	**0.5**
**accuracy**	0.782	0.76	0.759	0.654	0.762	0.705	0.628	0.758	0.726	0.662
**precision**	0.784	0.76	0.7608	0.6848	0.7632	0.7114	0.658	0.7595	0.7261	0.6638
**recall**	0.782	0.76	0.759	0.654	0.762	0.705	0.628	0.758	0.726	0.662
**f1**	0.7816	0.76	0.7586	0.639	0.7617	0.7028	0.6094	0.7577	0.726	0.6611

B.2 Task: Medical Condition

B.2.1 GPT4o

Table S4: Average metrics of GPT4o responses to perturbations

		Typographical			Homophone			Removal		
	**orig**	**0.1**	**0.3**	**0.5**	**0.1**	**0.2**	**0.3**	**0.1**	**0.3**	**0.5**
**accuracy**	0.7303	0.7333	0.7263	0.7092	0.7434	0.7223	0.7213	0.678	0.4507	0.0543
**precision**	0.9965	0.9988	0.9973	0.9975	0.9987	0.9976	0.9964	0.9961	0.9984	1
**recall**	0.7303	0.7333	0.7253	0.7092	0.7434	0.7223	0.7213	0.678	0.4507	0.0543
**f1**	0.8414	0.8446	0.8384	0.8281	0.8509	0.8363	0.8353	0.8052	0.6187	0.1024

B.2.2 LLama 3.1 8B

Table S5: Average metrics of LLama 3.1 8B responses to perturbations

		Typographical			Homophone			Removal		
	**orig**	**0.1**	**0.3**	**0.5**	**0.1**	**0.2**	**0.3**	**0.1**	**0.3**	**0.5**
**accuracy**	0.791	0.757	0.718	0.621	0.77	0.725	0.724	0.779	0.671	0.443
**precision**	0.834	0.823	0.814	0.784	0.832	0.81	0.818	0.833	0.782	0.714
**recall**	0.791	0.757	0.718	0.621	0.77	0.725	0.724	0.779	0.671	0.443
**f1**	0.8	0.772	0.739	0.644	0.783	0.743	0.745	0.79	0.676	0.423

B.2.3 BlueBERT

Table S6: Average metrics of BlueBERT responses to perturbations

		Typographical			Homophone			Removal		
	**orig**	**0.1**	**0.3**	**0.5**	**0.1**	**0.2**	**0.3**	**0.1**	**0.3**	**0.5**
**accuracy**	0.9279	0.9209	0.9239	0.9179	0.9139	0.8869	0.7868	0.9209	0.8959	0.7297
**precision**	0.9284	0.9214	0.9249	0.9193	0.9153	0.8933	0.8182	0.9218	0.8989	0.7562
**recall**	0.9279	0.9209	0.9239	0.9179	0.9139	0.8869	0.7868	0.9209	0.8959	0.7297
**f1**	0.9278	0.9206	0.9237	0.9175	0.9134	0.8856	0.7835	0.9207	0.8952	0.722

B.3 Task: Question and Answer

B.3.1 GPT4o

Table S7: Average metrics of GPT4o responses to perturbations

		Typographical			Homophone			Removal		
	orig	0.1	0.3	0.5	0.1	0.2	0.3	0.1	0.3	0.5
bleu	0.351	0.35	0.356	0.362	0.348	0.355	0.355	0.333	0.295	0.227
rouge	0.609	0.609	0.612	0.616	0.608	0.61	0.61	0.6	0.567	0.499
bert	0.632	0.633	0.638	0.643	0.631	0.635	0.634	0.621	0.591	0.509

B.3.2 LLama 3.1 8B

Table S8: Average metrics of LLama 3.1 8B responses to perturbations

		Typographical			Homophone			Removal		
	orig	0.1	0.3	0.5	0.1	0.2	0.3	0.1	0.3	0.5
bleu	0.314	0.312	0.309	0.304	0.307	0.305	0.297	0.297	0.241	0.164
rouge	0.52	0.525	0.527	0.523	0.518	0.521	0.516	0.512	0.462	0.372
bert	0.925	0.924	0.922	0.921	0.923	0.922	0.921	0.922	0.913	0.896

C. Experimental Settings

Setting	OpenAI	Llama	BlueBERT
Version	GPT4	Llama 3.1 8B	Bio_ClinicalBERT
Hardware	Google Colab Pro (T4 GPU)	Google Colab Pro (T4 GPU)	Google Colab Pro (T4 GPU)
Parameters	temperature: 1<br>top_p: 1<br>max_tokens: depends on task<br>stop: none<br>(other parameters): default	temperature: 0.7<br>top_p: 1<br>top_k: none<br>max_tokens: depends on task<br>(other parameters): default	num_train_epochs: 3<br>per_device_train_batch_size: 4<br>per_device_eval_batch_size: 4<br>warmup_steps: 10<br>weight_decay: 0.1

D. ANOVA test

To examine the relative impact of perturbations across dimensions RQ2, we conducted repeated measure ANOVA across tasks, perturbations, perturbation levels and LLMs in Microsoft Excel. This analysis was conducted using two factor ANOVA without replication and only the variations across columns were extracted from the analyses.

Then, depending upon the significance, we performed post-hoc pairwise t-tests with Bonferroni corrections. All t-test, except the highlighted two, were significant. This indicates that performance drop rather being a monolithic phenomenon, varies notably across perturbations, perturbation levels, tasks, and the choice of LLMs.

The results are shown in Table A-3 below:

Table S9. Repeated measure ANOVA and t-tests across dimensions showing that group variation for each dimension is significant. Post-hoc tests using Student's t-test with Bonferroni correction are conducted to examine pairwise variations. Other than two of the t-tests (highlighted t-tests are not significant), the pairwise t-tests are significant.

Groups (Repeated measure ANOVA)	t-test: Paired two sample for Means (with Bonferroni correction)				
	**Group1**	**Group2**	**t-stat**	**df**	**p-val**
**Perturbations**	Typographical	Homophone	-1.22	15	.08
	Homophone	Redaction	2.71	15	.0053
(F=9.9, DF=2,30, P=0.003)	Redaction	Typographical	-2.59	15	.0069
**Perturbation Levels**	Original	Low	3.38	23	.0009
	Low	Medium	3.78	23	.0003
(F=13.9, DF= 2, 46, P=0.00002)	Medium	Original	-3.73	23	.0004
**Tasks**	Sentiment	MedicalAbstract	-2.49	13	.009
	MedicalAbstract	QuestionAnswer	5.79	13	< .001
(F=6.0, DF=2,26, P=0.007)	QuestionAnswer	Sentiment	-0.931	13	.123
**LLMs**	GPT	BlueBERT	-7.66	13	< .001
	BlueBERT	Llama	2.44	13	.009
(F=27.39, DF=2,26, P=0.0000004)	Llama	GPT	4.27	13	.0003
